# Removal of Paracetamol from Aqueous Solutions Using Activated Carbon Obtained from Methane Pyrolysis-Derived Carbon Black

**DOI:** 10.3390/molecules31193496

**Published:** 2026-10-01

**Authors:** Matvey S. Galtsov-Tsientsiala, Artur A. Dzeranov, Alexander O. Dudoladov, Gulzhian I. Dzhardimalieva, Kamila A. Kydralieva, Elena A. Tarasova, Aleksey A. Leonov, Yaroslava V. Mikhailova, Ellina N. Burkova, Mikhail S. Vlaskin

**Affiliations:** 1 Joint Institute for High Temperatures of the Russian Academy of Sciences, 13/2 Izhorskaya St, 125412 Moscow, Russia; nerfangorn@gmail.com (A.O.D.); mailtarasovael@yandex.ru (E.A.T.); aleo03@yandex.ru (A.A.L.); yaroslava_ugansk@mail.ru (Y.V.M.); ellinaburkova04@mail.ru (E.N.B.); vlaskin@inbox.ru (M.S.V.); 2 Department of Materials Science, Moscow Aviation Institute (National Research University), Volokolamskoe Shosse, 4, 125993 Moscow, Russia; arturdzeranov99@gmail.com (A.A.D.); g_dzhardim@mail.ru (G.I.D.); k_kamila@mail.ru (K.A.K.); 3 Federal Research Center of Problems of Chemical Physics and Medicinal Chemistry, Russian Academy of Sciences, Semenov Avenue, 1, 142432 Chernogolovka, Russia

**Keywords:** paracetamol, methane pyrolysis, carbon black, pyrolytic carbon, activated carbon, CO_2_ activation, adsorption

## Abstract

Paracetamol (PCT), along with other pharmaceutical pollutants, increasingly requires removal from wastewater, whereas carbon black produced via methane pyrolysis can serve as a reliable and sustainable carbon source for the corresponding sorbents. This study evaluates the effect of CO_2_-based high-temperature activation parameters (temperature, duration, feed mass, composition of the activating gas) on the morphology, pore architecture, and PCT adsorption performance of activated carbon obtained from methane pyrolysis-derived carbon black. The sorption capacity of the activated carbon with respect to PCT, as well as the effect of initial pH (5–9) on adsorption isotherms and kinetics were studied. Among the investigated activation conditions (800–1000 °C, 60–180 min and different feed masses), the best-performing material exhibited a BET surface area of 712 m^2^·g^−1^ and a total pore volume of 0.44 cm^3^·g^−1^ (predominantly slit-shaped mesopores) and was obtained at 1000 °C for 180 min using a mass of 5.65 g. The resulting sorbent exhibits a PCT capacity of 629 mg·g^−1^ in a neutral medium and 718 mg·g^−1^ at pH 5. A physisorption mechanism with an adsorption energy of less than 8 kJ·mol^−1^ was shown. The kinetics of sorption obeyed a pseudo-second-order model with equilibrium within 5 min. The results demonstrated the potential of using activated carbon produced from carbon black (a product of methane pyrolysis) to remove pharmaceutical contaminants from water.

## 1. Introduction

Paracetamol (PCT), along with other pharmaceutical compounds [1], is increasingly being detected in various water bodies, including effluent from wastewater treatment plants, surface water, groundwater, and drinking water. This is due to its high resistance to biodegradation [2,3,4] and its ability to remain active in the environment. Such substances are classified as ‘contaminants of emerging concern’ [1,5,6], which pose a potential risk to human health and the environment even at trace concentrations [7,8]. During the COVID-19 pandemic, consumption of this drug increased by 50%, leading to a significant rise in its concentration in the environment [9,10]. Removing such contaminants from aquatic systems is a challenging task, as traditional biological treatment methods often fail to ensure their complete degradation. In this context, adsorption on carbon-based materials is considered an effective and economically viable method of post-treatment, with π–π interactions between the pollutant’s aromatic ring and the graphite-like structures of the sorbent’s surface playing a key role in retaining PCT molecules [11].

The development of effective water purification methods requires a detailed understanding of the relationship between the structure of a carbon adsorbent and its adsorption capacity. A key factor in this is the structural and morphological characterisation of the surface, which goes beyond traditional integral parameters such as specific surface area and pore volume. The classical description of porosity does not account for surface heterogeneity and roughness, which can significantly influence the material’s adsorption capacity with respect to organic pollutants. Approaches based on fractal geometry are increasingly being used to quantitatively describe this heterogeneity [12]. The fractal dimension (D) is one of the key characteristics for assessing the degree of irregularity and roughness of the surface of a porous solid [12]. A value of D = 2 corresponds to an ideally smooth surface, whilst a value approaching 3 reflects an extremely complex, ‘rough’ surface. The advantage of the fractal approach lies in its ability to describe the geometry of the pore space hierarchically, that is, to account for the self-similarity of the structure across a wide range of scales—from micropores (less than 2 nm) to mesopores (2–50 nm). Furthermore, fractal analysis can serve as a tool for predicting the efficiency of removing pollutants from aqueous media, in particular PCT. The literature demonstrates a link between the fractal dimension of porous carbon materials and their ability to remove synthetic dyes from aqueous solutions [13]. Thus, this approach opens new possibilities for understanding adsorption mechanisms and optimising the structure of carbon materials for water treatment purposes.

A promising carbon material for wastewater purification is a carbon black, obtained during methane pyrolysis. Hydrocarbon pyrolysis is a technology for producing hydrogen and carbon black without emitting carbon dioxide into the atmosphere, as shown by the following reaction equation:

CH_4_ → C↓ + 2H_2_↑

This process allows for the utilisation not only of methane, but also of associated petroleum gas, which is currently often flared during oil production, leading to atmospheric pollution and the wasteful consumption of valuable hydrocarbon feedstock [14,15]. The industrial implementation of this technology can improve the efficiency of natural resource use, whilst ensuring a zero-carbon footprint for the process, in contrast to traditional steam reforming of methane, which is associated with large-scale CO_2_ emissions [16]. Furthermore, identifying applications for the resulting pyrolytic carbon black plays a key role in enhancing the economic viability of the pyrolysis method [16].

One promising application of activated carbon is the removal of ecotoxicants, particularly pharmaceutical compounds, from wastewater [2]. To enhance sorption capacity, its surface can be modified to develop a highly porous structure through chemical [17] or physical activation methods [18]. Physical activation is considered environmentally safe, as it avoids the use of acids that would require disposal, unlike chemical activation [19]. Despite the longer activation time, physical activation allows sorption properties comparable to those achieved by chemical activation to be attained [20,21,22].

This work systematically elucidates the role of physical activation parameters (temperature, duration, feed mass, activator type) on the morphology, pore architecture, and PCT adsorption performance of activated carbon derived from pyrolytic carbon black. This study aims to (i) determine activation conditions for maximum specific surface area, (ii) evaluate PCT sorption capacity as a function of fractal dimensions, and (iii) establish correlations between structural complexity and adsorption efficiency. This study investigates the potential of waste-derived pyrolytic carbon for environmentally sustainable water purification.

## 2. Experimental Part

### 2.1. Materials

The raw material used was pyrolytic carbon black produced by the thermal decomposition of methane in an oxygen-free environment within a corundum tube at a process temperature of 1200 °C and a flow rate of 1 L·min^−1^, as described in [23]. The purity of the pyrolytic carbon black was 99.95%, and the particle size ranged from 157 to 896 nm. Grade 5.0 nitrogen (99.999% by volume) was used to purge the tube to remove air. Physical activation was carried out using CO_2_ (grade ‘Premium’, UN No. 1013 (UN1013)) as the activating reagent. Both gases were produced at the Moscow Gas Processing Plant. The experiments were conducted in a ceramic tube measuring 60 mm × 50 mm × 1000 mm. Al_2_O_3_ content ≥ 99%, density ≥ 3.85 g·cm^−3^. For the sorption experiments, PCT (active ingredient: acetaminophen, 500 mg per tablet) from Kirkland (Brentwood, TN, USA) was used. White filter paper and MCE 0.22 µm syringe filters were used to filter the PCT and sorbent solutions.

### 2.2. Methodology for Producing Activated Carbon

**Figure 1:**
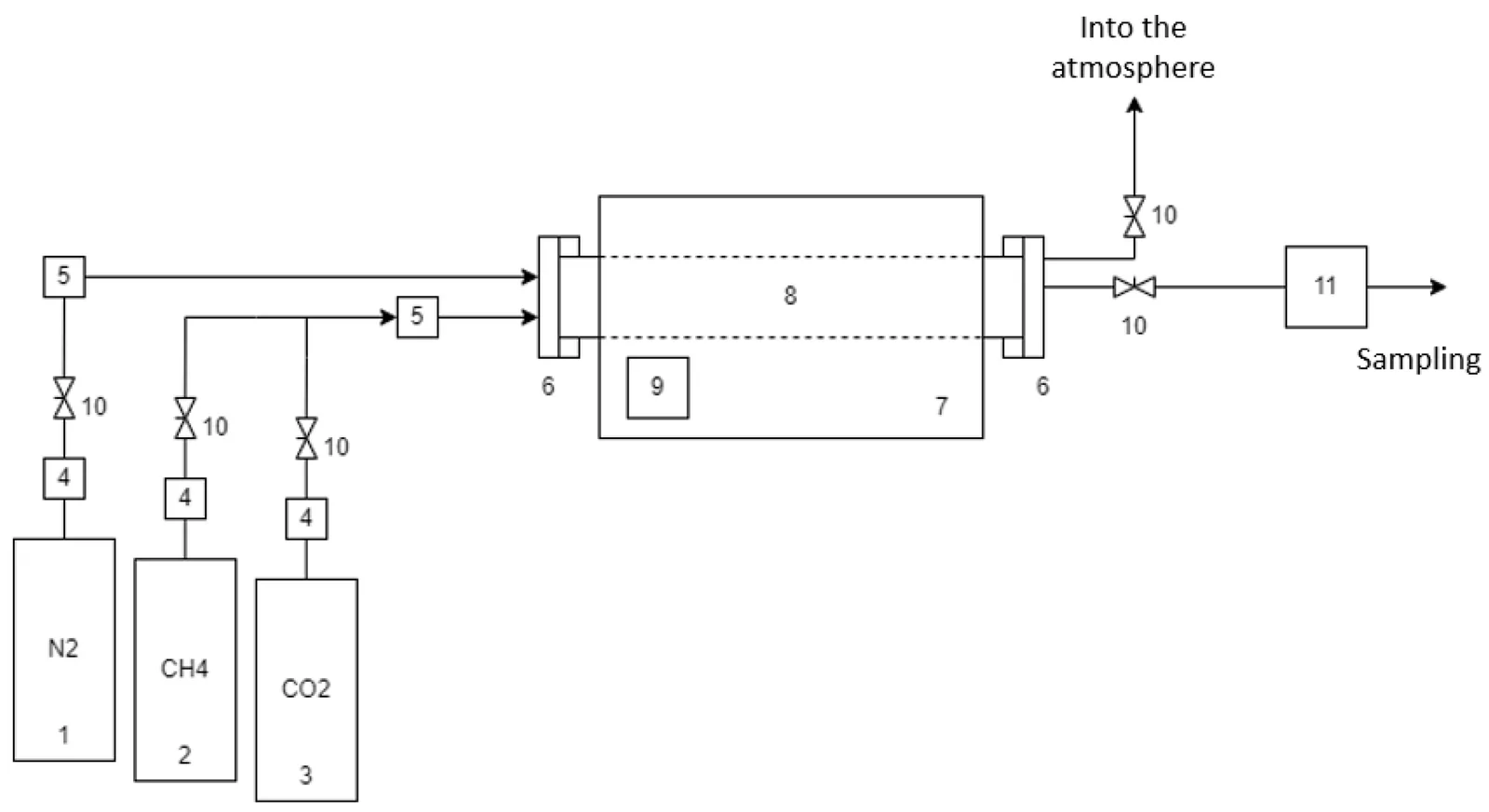
Scheme of the experimental setup for methane pyrolysis and the activation of pyrolytic carbon black: 1—nitrogen cylinder, 2—methane cylinder, 3—carbon dioxide cylinder, 4—reducers, 5—rotameters, 6—flanges, 7—oven, 8—corundum tube, 9—control unit, 10—needle valves, 11—filter.

Methane pyrolysis and the activation of pyrolytic carbon black were carried out in a tubular furnace, a diagram of which is shown in Figure 1.

The experimental setup consists of a XINYU (Nanyang, China) SK2-6-17TPB4 tubular furnace with a capacity of 6 kW. A corundum tube with an internal diameter of 50 mm is installed inside the furnace. The furnace heaters are made of molybdenum disilicide, and the heating zone is 440 mm long. The furnace is equipped with a temperature controller. Nitrogen and carbon dioxide are fed into the reactor from tanks via a pressure reducer, with the flow rate controlled by a rotameter. Gas-tight rubber gaskets are used as seals in the flanges.

Before starting the furnace, the carbon black collected after pyrolysis (1200 °C, flow rate 1 L/min [23]) was ground in a mortar and placed into a ceramic boat (crucible), which was positioned in the central zone of the reactor. Once the set temperature was reached, the nitrogen supply was switched to carbon dioxide. The experiment is carried out for a set duration, after which the furnace is switched off and purged with nitrogen. The boat containing the pyrolytic carbon black is removed from the tube and weighed on a balance. Activation experiments were conducted at temperatures of 800–1000 °C and a carbon dioxide flow rate of 0.1 L/min.

### 2.3. Adsorption Experiment Methodology

Sorption experiments were carried out in 100 mL conical flasks, to which 40 mL of a solution containing a specified concentration of PCT (0–500 mg·L^−1^) was added. The sorbent was then suspended at a concentration of 1 g·L^−1^, and the solution was mixed on an orbital shaker (250 rpm, 24 h, room temperature). Sorption kinetics were studied at time intervals of 5, 10, 30, 60, and 180 min and 22 h at a constant PCT concentration of 500 mg·L^−1^. After sorption, the sorbent was separated from the solution using filters (white strip and 0.22 µm syringe filters). Due to the limited amount of synthesised sorbent material, independent replicate adsorption experiments were not performed; therefore, the reported values represent individual experimental measurements.

The sorption isotherms were fitted using the Langmuir [24], Freundlich [25], and Dubinin–Radushkevich [26] models.

The Langmuir model assumes monomolecular adsorption on a homogeneous surface:

q_e_ = (q_max_·K_L_·C_e_)/(1 + K_L_·C_e_),

Linearised form:

C_e_/q_e_ = 1/(K_L_·q_max_) + C_e_/q_max_,

where q_e_ is the equilibrium adsorption capacity (mg·g^−1^), q_max_ is the maximum adsorption capacity (mg·g^−1^), K_L_ is the Langmuir constant (L⋅mg^−1^), and C_e_ is the equilibrium concentration of the adsorbate (mg⋅L^−1^).

The Freundlich empirical adsorption model on an energetically heterogeneous surface:

q_e_ = K_F_·C_e_^1/n^,

Linearised form:

ln(q_e_) = ln(K_F_) + 1/n·ln(C_e_),

where K_F_ is the Freundlich constant (mg⋅g^−1^), and n is the adsorption intensity parameter.

The Dubinin–Radushkevich (DR) model is used to describe adsorption in microporous materials:

q_e_ = q_m_·exp(−βε^2^),

where q_m_ is the theoretical maximum adsorption capacity (mg⋅g^−1^), β is a constant related to the adsorption energy (mol^2^⋅J^−2^), and ε is the Polanyi potential (J⋅mol^−1^).

The sorption kinetics data were fitted using pseudo-first-order [27], pseudo-second-order [28], and intraparticle diffusion [29] models.

The differential form of the pseudo-first-order model:

(dq_t_)/dt = k_1_·(q_e_ − q_t_),

Linearised form:

ln(q_e_ − q_t_) = ln(q_e_) − k_1_·t

where q_t_ is the amount of adsorbed substance at time (t, min) (mg⋅g^−1^), and k_1_ is the pseudo-first-order rate constant (min^−1^).

The differential form of the pseudo-second-order model:

(dq_t_)/dt = k_2_· (q_e_ − q_t_)^2^,

Linearised form:

t/q_t_ = 1/(k_2_·q_e_^2^) + t/q_e_

where k_2_ is the pseudo-second-order rate constant (g⋅mg^−1^⋅min^−1^).

The intraparticle diffusion model is used to assess the effect of diffusion within the pores on the adsorption kinetics:

q_t_ = k_id_·t^1/2^ + C,

where k_id_ is the rate constant for intraparticle diffusion (mg⋅g^−1^⋅min^−1/2^), and C is a constant related to the thickness of the boundary layer (mg⋅g^−1^).

### 2.4. Characterisation Methods

#### 2.4.1. Specific Surface Area (BET)

The textural characteristics of the samples were determined from nitrogen adsorption isotherms at 77 K, recorded using a Nova 2200e surface area analyser (Quantachrome, Boynton Beach, FL, USA). Prior to isotherm measurement, the samples were degassed at 120 °C and a residual pressure of 10^–3^ mm Hg for 3 h. The specific surface area of the materials based on physical adsorption isotherms was determined using the BET (Brunauer-Emmett-Teller) equation:

P/(V(P_0_ − P)) = 1/(V_m_·C) + (C − 1)/(V_m_·C)∙P/P_0_,

where P is the equilibrium pressure of the adsorbate, P_0_ is the saturated vapor pressure, V is the volume of the adsorbed gas, V_m_ is the volume of the monomolecular layer, and C is the BET constant.

#### 2.4.2. Total Pore Volume

The total pore volume was determined using the Dubinin–Radushkevich equation and the nitrogen adsorption isotherm at a relative pressure of 0.995:

W = W_0_·exp(−(A/E_0_)^2^),

where W is the volume of adsorbate filling the micropores, W_0_ is the total micropore volume, A is the adsorption potential, and E_0_ is the characteristic adsorption energy.

#### 2.4.3. Pore Size Distribution

The pore size distribution was determined using the BJH (Barrett–Joyner–Halenda) method based on the desorption isotherm:

ln(P/P_0_)= −(2γV_m_)/(r_k_·RT),

where γ is the surface tension, V_m_ is the molar volume, r_k_ is the radius of curvature of the meniscus, R is the universal gas constant, and T is the temperature.

#### 2.4.4. Raman Spectroscopy

Raman spectroscopy was used to characterise the structural ordering and defect-related features of the activated carbon. Raman spectra were recorded using a Thermo Scientific (Waltham, MA, USA) DXR Raman Microscope, with a 532 nm excitation laser at 3 W.

#### 2.4.5. Spectrophotometry

The equilibrium concentrations of PCT in the supernatant were determined by UV-visible spectroscopy on a Solar (Minsk, Belarus) UV-Vis spectrophotometer using calibration curves for PCT. Measurements were performed at a wavelength of 243 nm. For each experiment, a calibration curve was generated using PCT concentrations of 1.5625, 3.125, 6.25, 12.5, 25, and 50 mg L^−1^, with a coefficient of determination of R^2^ = 0.9997. A blank sample was used during spectrophotometric data acquisition to subtract the background signal.

## 3. Results and Discussion

### 3.1. Mass Loss of Pyrolytic Carbon Black During Activation

The search for optimal conditions for the physical activation of pyrolytic carbon black in terms of specific surface area (SSA) was carried out by varying the activation conditions: feed mass, temperature, process duration, and type of activator (Table 1). The physical activation of pyrolytic carbon black was accompanied by a change in the sample mass depending on the activation process parameters.

The samples with the lowest backfill mass lost the greatest proportion of mass (42.4%, 51.64%, and 35.04%). The smallest loss was observed in pyrolytic carbon black activated at 800 °C and with a high loading mass. The exception was sample S5, which, although it had a low loading mass, showed mass losses comparable to those of sample S2, whose mass was twice as high. This is due to the fact that sample S5 contained a large amount of carbon film (formed during the pyrolysis of carbon black, as shown in [23]), which has a denser and stiffer structure compared to powdered carbon.

### 3.2. The Physicochemical Properties of Activated Carbon

#### 3.2.1. The Low-Temperature Nitrogen Adsorption–Desorption Isotherms

**Figure 2:**
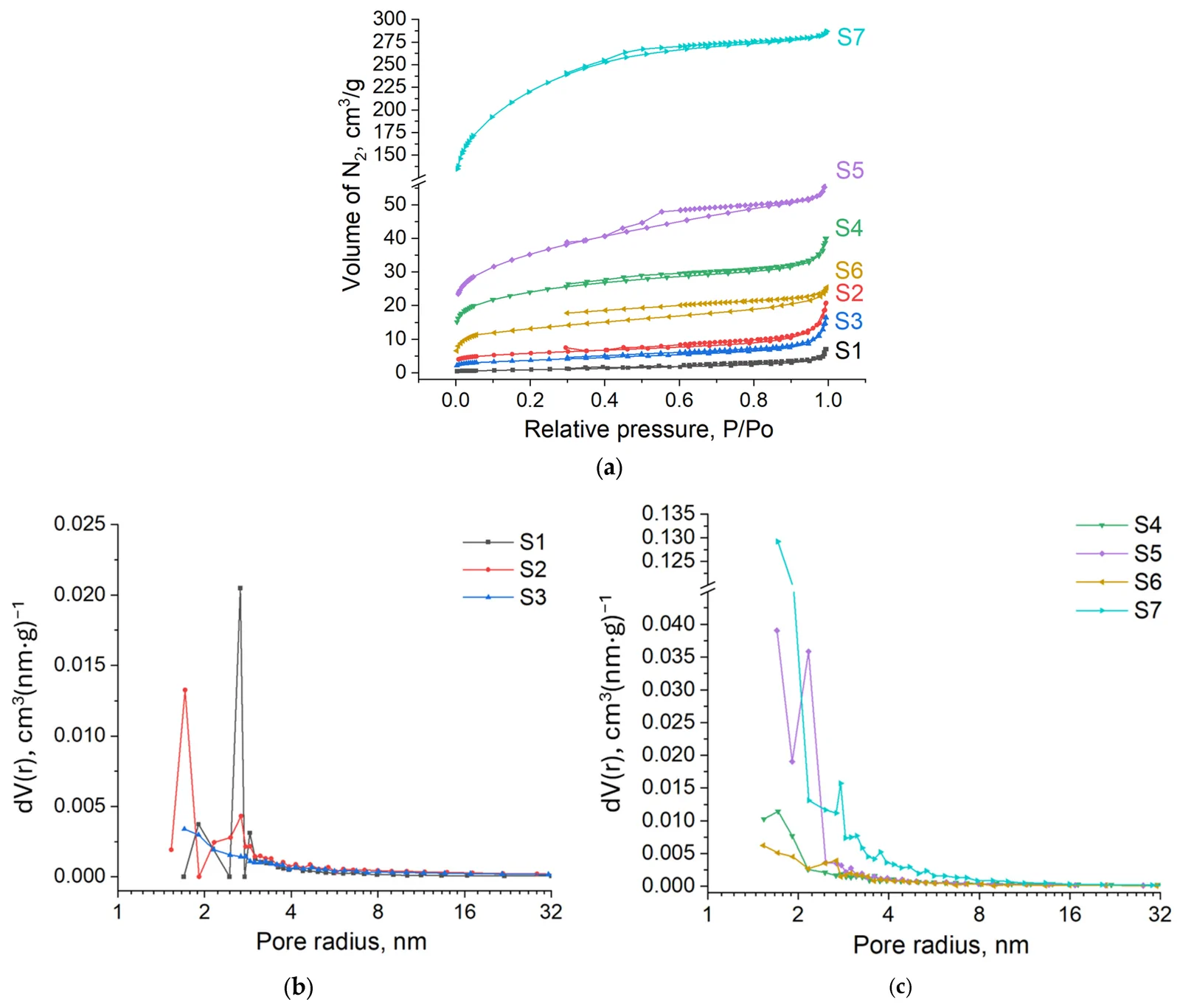
Nitrogen adsorption/desorption isotherms (**a**) and pore size distribution (**b**,**c**) for activated carbon samples.

Figure 2 shows the low-temperature nitrogen adsorption–desorption isotherms for sorbent samples S1–S7 obtained under various physical activation conditions. All isotherms belong to type IV(a) according to the IUPAC classification [30], which is characteristic of mesoporous materials with a developed SSA. In the initial section of the isotherms (P/P_0_ < 0.1), a steep rise is observed, indicating the presence of a significant volume of micropores, which are filled at low relative pressures.

All samples exhibit a hysteresis loop, the shape of which varies depending on the activation conditions. Samples S1–S3 show an H3-type loop, corresponding to open pores between particles. For samples S4–S7, activated at higher temperatures and with prolonged holding times, the hysteresis loop shifts towards the H4 type, characterised by a steep rise on the isotherm at low pressures, indicating a high contribution to porosity from micropores [31]. The presence of hysteresis indicates the phenomenon of capillary adsorption in mesopores [31] (Figure 2). Types H3 and H4 are found in materials with slit-shaped pores [31]. The highest volume of adsorbed nitrogen, corresponding to the maximum SSA (712.8 m^2^·g^−1^), was recorded for the sample activated at 1000 °C for 180 min in a CO_2_ atmosphere (initial mass 5.65 g) (Table 2).

#### 3.2.2. Frenkel–Hosley–Hill Model

To quantitatively assess the topological heterogeneity of the surface of the synthesised materials, the Frenkel–Holsey–Hill (FHH) model was used, based on an analysis of nitrogen adsorption isotherms in the capillary condensation region (Figure 2a). The geometric complexity of the surface structure of many solids can be characterised by the fractal dimension D. Values of D range from 2 to 3 and express the degree of complexity of the surface and/or porous structure [32]. The FHH model describes multilayer adsorption on a heterogeneous surface, taking into account the interaction potential, which decreases with distance. In the case of activated carbons, the following form of the equation is often used [33]:

ln(V) = C + A·ln(ln(P_0_/P)).

where V is the volume of the adsorbed gas at equilibrium pressure P, P_0_ is the saturation pressure of the gas at a given temperature, C is the pre-exponential factor, and A is the exponent of the power law, which depends on D and the adsorption mechanism.

The classical FHH model assumes that the adsorption potential is independent of pore size. In micropores (<2 nm), the adsorption potentials from opposite walls overlap, leading to a significant overestimation of D_ads_. For physically activated carbons, the standard approach is to split the fractal dimension into two parameters [34], which are calculated from different linear segments on the FHH plot within the range P/P_0_ = 0.2–0.8 (Figure 2a). The surface fractal dimension D_ads1_ (local roughness of pore walls; P/P_0_ = 0.2–0.5), characterising the roughness of micropore walls and the degree of heterogeneity of the adsorption potential in narrow pores. The fractal dimension of the structure D_ads2_ (mesopores, P/P_0_ = 0.5–0.8), reflecting the complexity of the spatial organisation of mesopores, the tortuosity of the channels, and the roughness of the walls of larger pores.

In this case, [35] applies:

D = 3 + A

The D value obtained from the FHH model provides important information about the structure of the coal:

- D = 2.0—smooth surface (e.g., graphitised carbon black or coal with predominantly slit-like micropores and no roughness).
- 2.0 < D < 2.5—moderately rough surface. Typical of coals activated by steam or CO_2_ with a low degree of charring.
- 2.5 < D < 3.0—highly developed, fractal surface. Typical of chemically activated coals, where a developed mesoporous structure with highly rough pore walls is formed.
- D = 3.0 (or >3)—an extreme case, meaning that the surface is so rough that it fills the volume (‘porosity without a smooth surface’), or that the model’s scope of applicability has been exceeded.

The D values range from 2.36 to 2.98, which is typical for porous materials with a highly irregular surface (fractal dimension between 2 and 3). High coefficients of determination (R^2^ > 0.96 in most cases) confirm the applicability of the FHH model (Table 3). For all samples, D_ads2_ > D_ads1_, which is typical: mesopores generally exhibit greater surface heterogeneity and fractal complexity, whereas micropores tend to possess a more uniform surface structure. The greatest difference is observed in sample S1 (D_ads1_ = 2.36 and D_ads2_ = 2.60) and sample S7 (D_ads1_ = 2.78 and D_ads2_ = 2.96), indicating a significant difference in roughness between micropores and mesopores. The smallest difference is observed in sample S2 (D_ads1_ = 2.74 and D_ads2_ = 2.81) and sample S4 (D_ads1_ = 2.83 and D_ads2_ = 2.93), which indicates a more homogeneous (self-similar) fractal structure.

Understanding how fractal dimensions vary with activation parameters allows for the targeted control of coal properties. Studies of the steam activation of bituminous coal at various temperatures have shown [34] that mesopores develop more effectively at 750 °C. The D_ads2_ value (structural fractality) is higher, indicating the formation of a complex, branched network of mesopores. This is characteristic of a gentle treatment regime, where carbon burnout proceeds uniformly throughout the volume. At 850 °C, the process intensifies, but some mesopores may ‘collapse’ or coalesce due to wall thickening. D_ads2_ may decrease, whilst microporosity (D_ads1_) reaches a maximum. The degree of charring is a key parameter of physical activation. As the degree of charring increases, D_ads1_ (microporous surface area) initially increases due to the formation of new micropores, and the walls become rough. Then, at very high degrees of charring, it begins to decrease due to the expansion of micropores, which transition into the mesopore category, and their ‘roughness’ is smoothed out. D_ads2_ (mesopore structure) tends to increase as the degree of charring increases. The burning away of the partitions between the pores creates a more complex fractal network of interconnected channels.

**Figure 3:**
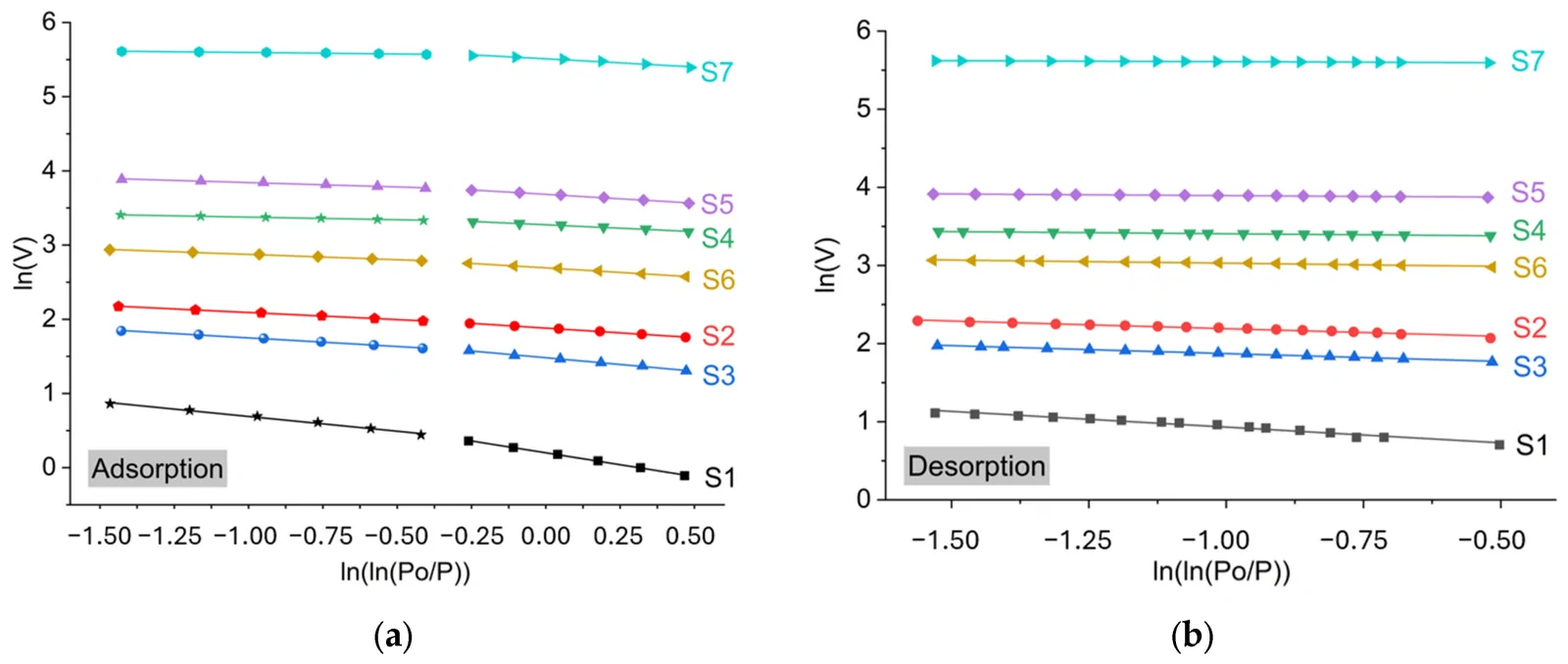
Plots of ln(V) versus ln(ln(P_0_/P)), reconstructed from the N_2_ adsorption (**a**) and desorption (**b**) isotherms.

For activated carbons, it is common to analyse not only the adsorption branch, but also the desorption branch of the isotherm. For the desorption branch, a different mechanism is typically employed: not multilayer adsorption, but capillary condensation. Although the classical FHH model was originally developed for multilayer adsorption, it is also commonly applied to the desorption branch for comparative fractal analysis of porous materials in the capillary condensation regime [35]. In this case, the calculated D_des_ values reflect the geometric complexity of the pore network rather than a strict surface fractal dimension. Analysis of the desorption branch in the P/P_0_ range from ~0.85 to ~0.5 allows the fractal dimension of the pore surface to be characterised in the capillary condensation regime (Figure 3b). In this regime, the ‘liquid–vapour’ interface forms inside the pores, and its shape is determined by the geometry of the pore space. According to the data obtained, samples S1, S2, S3, S4, and S7 exhibit similar values of D_des_ and D_ads2_ (difference ≤0.03) (Table 3). This suggests that hysteresis is weak, the pore space has a simple shape (e.g., cylindrical or slit-like pores without ‘bottle necks’), and capillary condensation during desorption is in good agreement with multilayer adsorption on mesopores. Samples S5 and S6 show a significantly higher D_des_ compared to D_ads2_, which is indicative of network effects and the presence of ‘ink bottle’-type pores (narrow neck–wide cavity). Such a structure hinders desorption, leading to an increase in the apparent fractal dimension calculated from the desorption branch. According to a study by Morlay and Joly [36], for commercial activated carbons (such as Filtrasorb 400), fractal analysis of the desorption branch yields D values in the range 2.7–2.9, indicating a highly developed, fractal pore structure, as determined by SEM.

#### 3.2.3. Raman Spectroscopy

**Figure 4:**
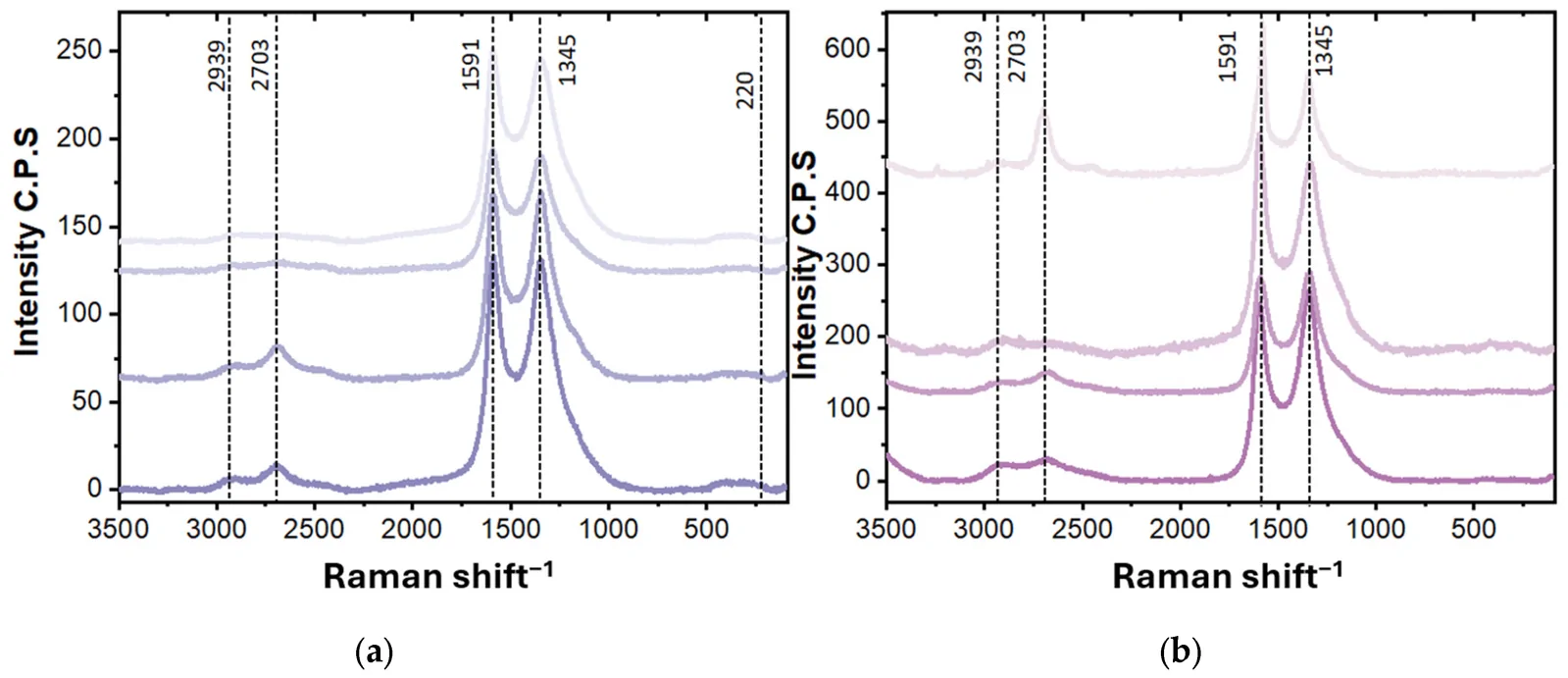
Combination scattering spectra of samples S1 (**a**) and S6 (**b**).

Raman spectroscopy was carried out to characterise the structural features of the carbon materials and to compare their degree of structural ordering/disorder. Figure 4 shows the Raman spectra of the samples for comparison with those of conventional activated carbon.

Raman spectra were recorded for samples S1 and S6 (Figure 4). Four Raman spectra were recorded at different points on each sample to assess the reproducibility and spatial homogeneity of the carbon material. Both samples exhibit peaks at 1345, 1591, 2703, and 2939 cm^−1^. These peaks correspond to the so-called G, D, and 2D regions [37].

**Figure 5:**
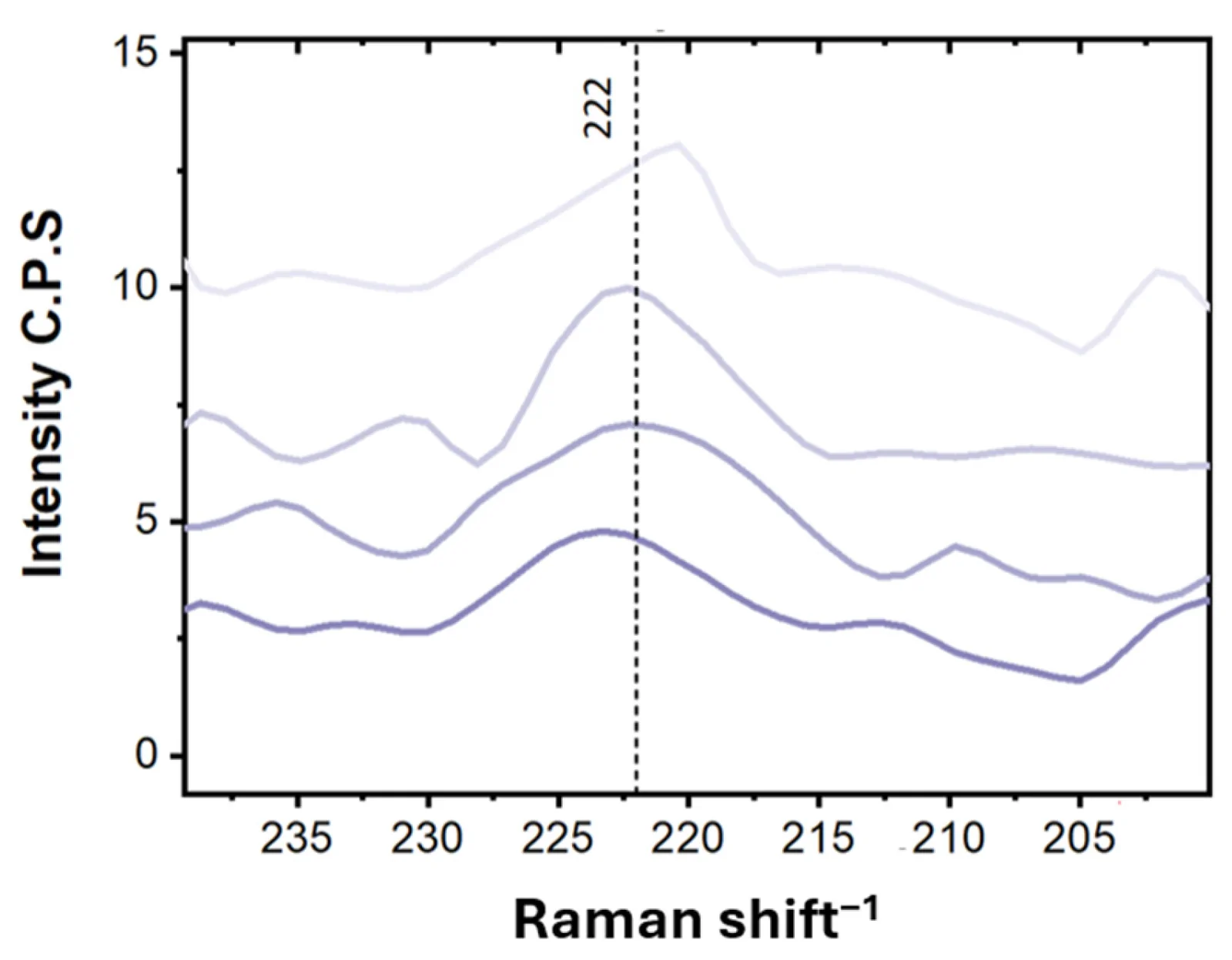
Raman spectra for sorbent S1 in the 236–205 cm^−1^ range.

A regular, low-intensity peak at 222 cm^−1^ is present in sample S1 (Figure 5). Its presence indicates the existence of an impurity. It is difficult to determine the nature of this impurity from a single low-intensity peak.

This low-intensity peak may indicate the presence of organic impurities that did not evaporate during the carbonisation process. Sample S1, in which this peak is observed, was activated at a temperature of 800 °C and for half the duration of the activation process, unlike sample S5.

### 3.3. Adsorption Efficiency

#### 3.3.1. Sorption Isotherms

**Figure 6:**
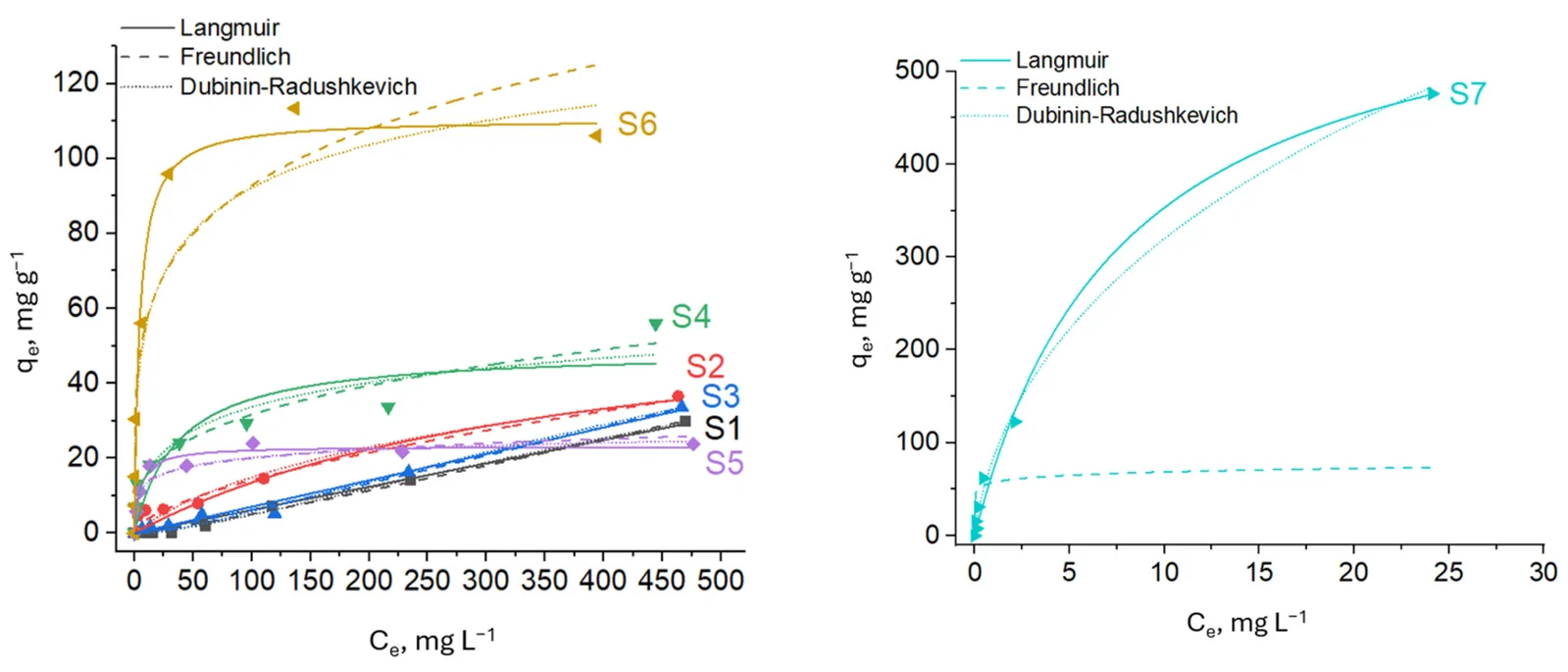
Sorption isotherms of PCT on activated carbon samples S1–S7 (C_sorbent_ = 1 g·L^−1^, 30 s ultrasound, 24 °C, 250 rpm, 24 h).

In order to assess the potential for using the activated carbon samples obtained as sorbents for ecotoxicants in aquatic environments, experiments were conducted to determine the sorption capacity of the activated carbons with respect to PCT, which was selected as a model contaminant. The PCT sorption isotherms in deionised water are shown in Figure 6. The sorption isotherms were approximated using the Langmuir, Freundlich and Dubinin–Radushkevich (DR) models.

The adsorption isotherms of samples S1–S4 were reasonably described by the Freundlich model, whereas the Langmuir model provided a particularly good description for samples S5–S7 (Table 4). The differences in the relative quality of the two models may reflect changes in the surface and pore structure associated with activation. However, these fitting results should not be interpreted as definitive evidence of a transition from a heterogeneous to a homogeneous surface. The heterogeneity parameter *n* varied among samples S1–S4, with values of 0.88, 1.66, 0.91, and 3.17, respectively. For effective sorbents, *n* is significantly greater than 1 (*n* > 1 indicates that the isotherm is convex and the adsorbate interacts with the surface more strongly than with the solvent) [25]. Higher *n* values have been associated with a more uniform distribution of adsorption energies [38]. However, the variation in *n* among S1–S4 is not strictly monotonic and therefore does not by itself demonstrate a progressive decrease in surface heterogeneity. The theoretical basis of the DR model [39] is Polanyi’s theory of pore volume filling, which assumes that sorption occurs predominantly through the filling of the micropore volume rather than the formation of a monolayer on the external surface. The main advantage of the DR model is that it allows estimation of the mean sorption energy (E), which can be used to assess the nature of the sorption process. A value of E in the range of 1–8 kJ mol^−1^ is generally associated with predominantly physical sorption, characterised by weak van der Waals interactions. The value of E for all samples does not exceed 8 kJ mol^−1^, supporting the predominantly physical nature of the sorption process rather than the formation of stronger chemical bonds, such as ion-exchange or covalent bonds [40]. This type of sorption is reversible and is often characterised by low energy requirements, which may be a favourable factor for the regeneration of the sorbent for reuse.

The observed difference in the experimental data describing the PCT adsorption isotherms of the samples is primarily related to the samples’ SSA. Samples with a smaller surface area exhibit behaviour characteristic of an energetically heterogeneous surface. This means that adsorption on them occurs primarily at the most active sites (e.g., defects or terminal functional groups), which is described by the Freundlich model. The less developed the surface, the more pronounced its heterogeneity becomes.

**Figure 7:**
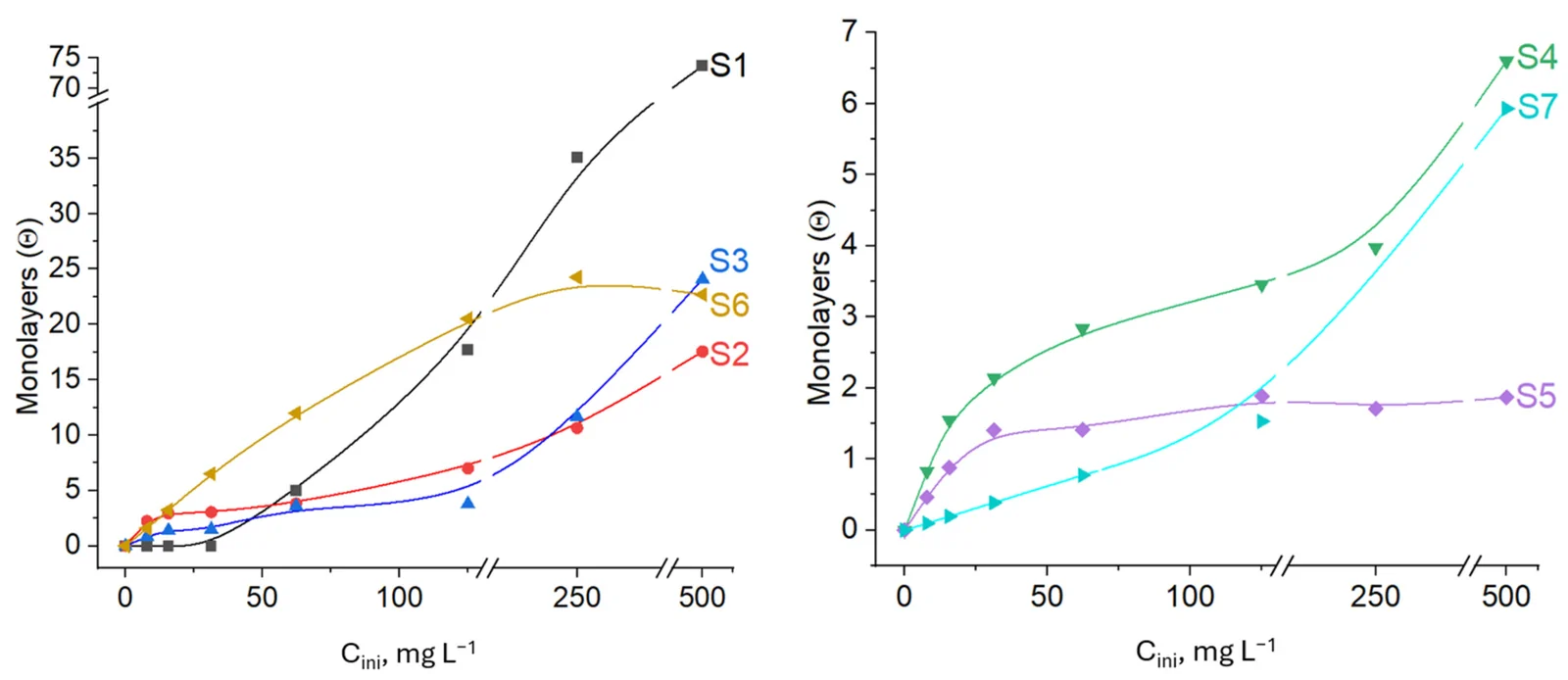
Equivalent surface monolayers (Θ) of PCT adsorption by activated carbon samples as a function of initial concentration (24 h, deionised water).

For samples with a high SSA, such as S7 (712 m^2^·g^−1^), the Langmuir model predominates. This suggests that activation results in a more uniform surface with a uniform distribution of adsorption sites, where π–π interactions may contribute to PCT adsorption [41]. Thus, it can be assumed that PCT adsorption in the case of samples with low SSA occurs predominantly not in the form of monolayers. Based on the assumption that, upon interaction with the sorbent, the entire surface area of the PCT molecule σ (2.2296 × 10^−18^ m^2^ molecule^−1^ [42]) occupies the adsorption sites, the number of equivalent surface monolayers (Θ) was calculated for all sorbents (Figure 7) using the formula

Θ = (0.001q_e_·Nσ)/(M_w_·SSA),

where N (6.02214076 × 10^23^): Avogadro’s number; Mw (g·mol^−1^): molecular weight of the sorbate; SSA (m^2^·g^−1^): specific surface area of the sorbent; σ (2.2296 × 10^−18^ m^2^·molecule^−1^): surface area of the sorbate molecule.

For all samples (except S5), the calculated value of Θ is significantly greater than 1. This indicates that the adsorption of PCT is not limited to a monomolecular layer. For most samples, Θ values significantly exceeded unity, suggesting that adsorption is not limited to the formation of a single molecular layer. This behaviour may be associated with multilayer adsorption, pore filling effects, and/or intermolecular interactions between adsorbed PCT molecules, particularly in samples with a highly developed porous structure.

#### 3.3.2. The Effect of Initial pH on Adsorption Isotherms and Kinetics

**Figure 8:**
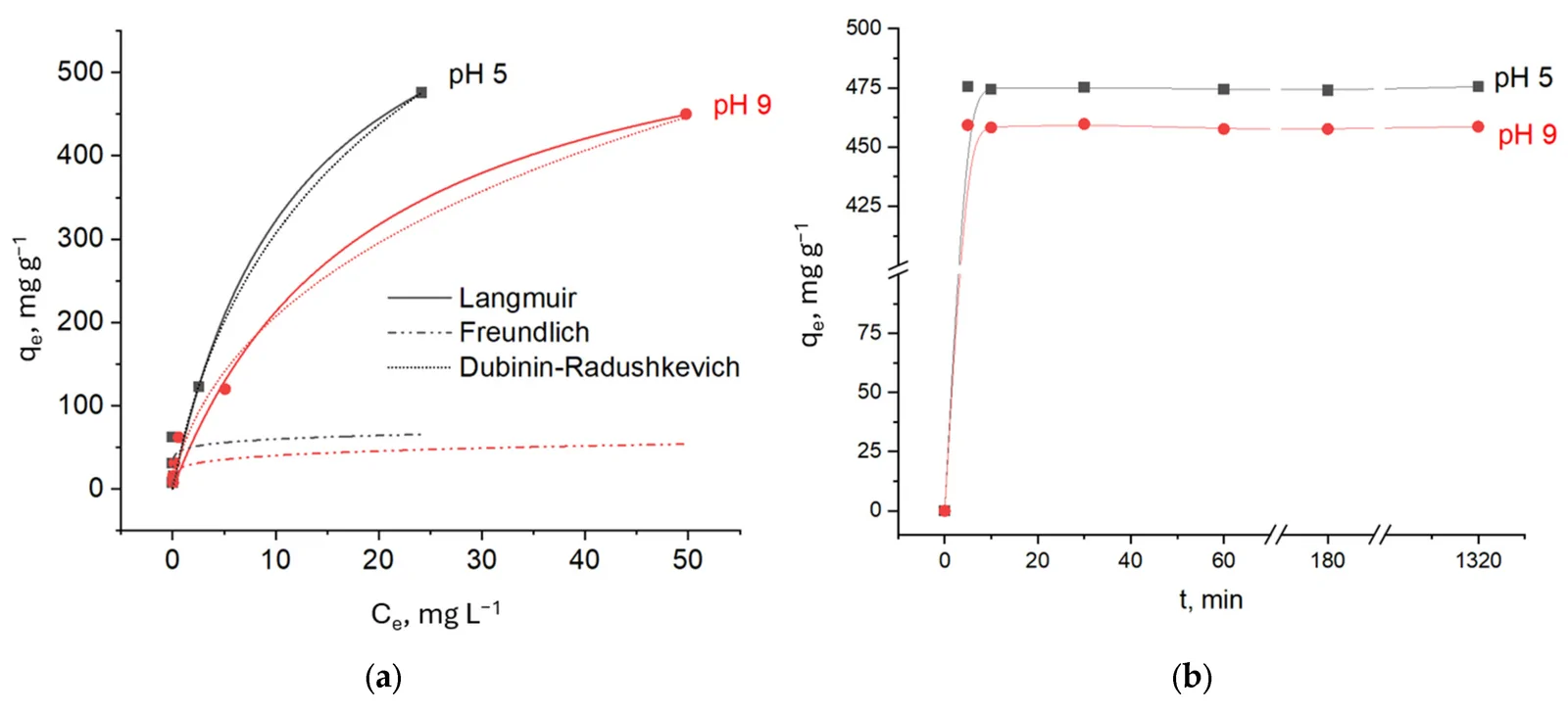
Isotherms (**a**) and kinetics (**b**) of PCT sorption by activated carbon at different pH values (C_sorbent_ = 1 g·L^−1^, 30 s ultrasound, 24 °C, 250 rpm).

To assess the effect of initial pH on adsorption isotherms and kinetics, PCT solutions with pH values of 5 and 9 (adjusted using 0.1 mol⋅L^−1^ HCl and NaOH) were selected. The studies focused on conditions typical of natural environments—water bodies and soils—where pH values lie exclusively within the range of 5 to 9 and do not exceed these limits. The most promising sample, S7, with a maximum SSA of 712 m^2^⋅g^−1^, was used as the sorbent. Figure 8a shows the adsorption isotherms in media with different pH values.

Based on Langmuir parameters, it was found that the materials exhibited high maximum adsorption capacities of 718 and 624 mg·g^−1^ at pH 5 and 9, respectively (Table 5). The carbon sorbent with the closest adsorption capacity values was investigated in the work by Agustín Spaltro et al. [43], where the authors reported q_max_ = 560 mg·g^−1^ at pH 3 for commercial CAT charcoal. For activated carbon derived from cotton fabric, obtained by chemical activation (H_3_PO_4_), in the work by Nassima Boudrahem et al. [44], q_max_ is 105 mg·g^−1^, whilst in the study by Wondimu Kebede Wakejo et al. [45], chemically activated (KOH/FeCl_3_) charcoal based on bamboo sawdust exhibited q_max_ = 188.76 mg·g^−1^. The results from the sorption capacity assessment show that the sample of pyrolytic carbon black obtained by the physical method outperforms many samples obtained by chemical activation and is comparable to the best commercial analogues. The E value for all samples does not exceed 8 kJ·mol^−1^, indicating physical sorption without the formation of stronger chemical bonds. At pH 5, the KL value (0.08) is higher than at pH 9 (0.05), which indicates a higher affinity of the adsorbent for PCT molecules at pH 5 and is consistent with literature data on the influence of pH on adsorption in studies of commercial activated carbon CAT [43]. The decrease in KL at pH 9 is probably due to a change in the hydration or aggregation of PCT, since at pH 9 it is predominantly in a neutral form (not dissociated) (pKa ~ 9.5).

**Figure 9:**
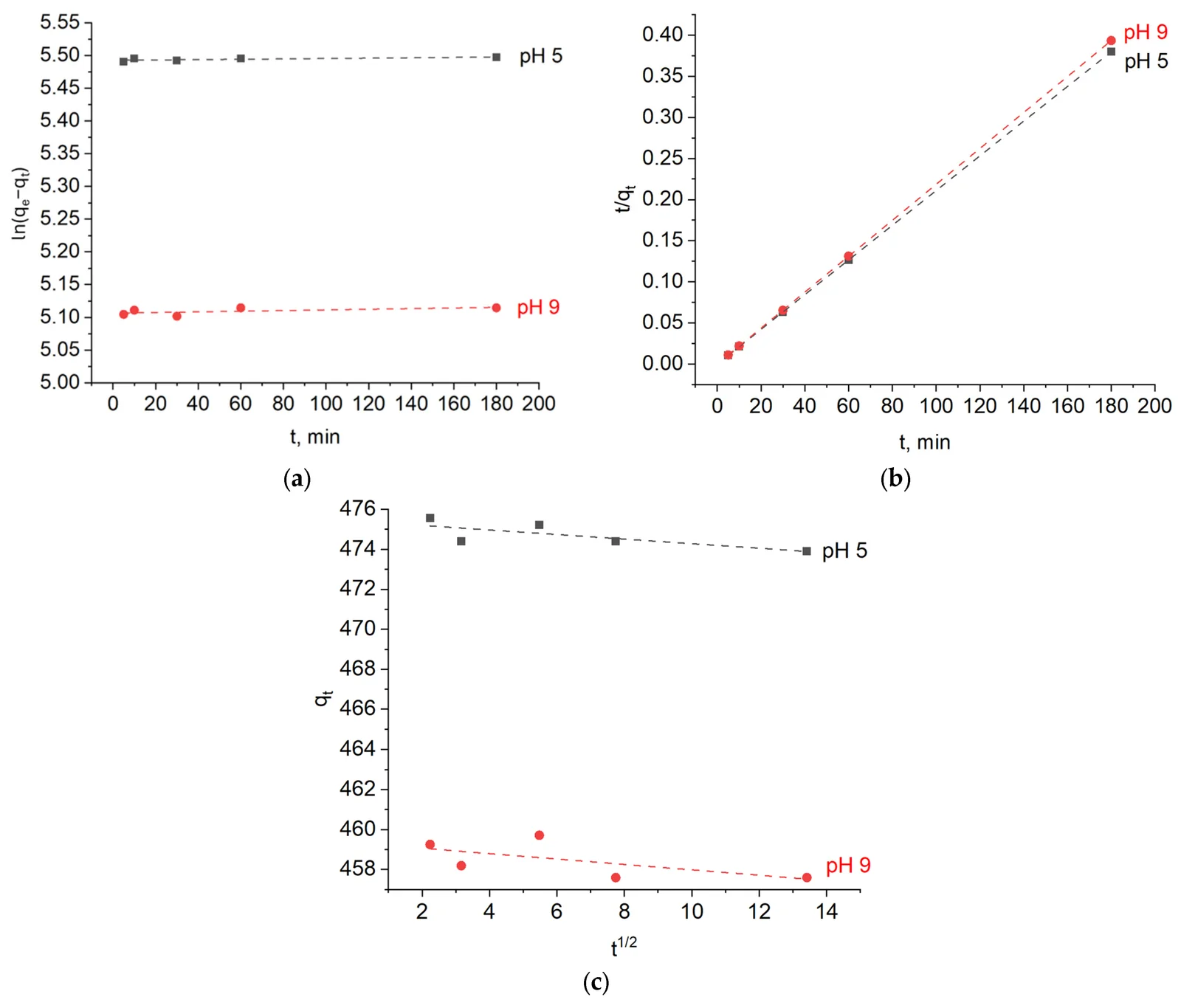
Linear forms of the pseudo-first- (**a**) and pseudo-second-order models (**b**) and the intraparticle diffusion model (**c**) (C_PCT_ = 500 mg L^−1^, C_sorbent_ = 1 g L^−1^, 30 s ultrasound, 24 °C, 250 rpm, 0–180 min).

The kinetic sorption curves for PCT using activated carbon at various pH values show that sorption equilibrium is reached rapidly (5 min) (Figure 8b). The majority of the sorbate is adsorbed within the first few minutes of contact between the sorbent and the solution, after which the sorption capacity remains virtually unchanged. This indicates the high availability of adsorption sites and the well-developed porous structure of the adsorbent, as mentioned above. Figure 9 presents an approximation of the PCT sorption kinetics data for the S7 adsorbent using linearised pseudo-first-order (PFO) [27], pseudo-second-order (PSO) [28], and intraparticle diffusion (IPD) models. The PFO equation describes the patterns of sorption during the initial stages of the sorption process, when the phenomenon of film diffusion has a significant influence on the process. The PSO equation allows for the consideration not only of sorbate–sorbent interactions, but also of intermolecular interactions between the sorbates. The IPD model assumes that the diffusion of the sorbate within the sorbent is the rate-limiting step of the process.

The linearised PSO model yielded the highest R^2^ values among the linearised kinetic models considered (Table 6), and the calculated q_e_ values were close to the experimental values. Thus, within the linearised analysis, the PSO equation provided a closer empirical description of the experimental kinetic data than the PFO equation. Nonlinear fitting of the PFO and PSO equations was also performed (Table 7). Both nonlinear models provided similarly close representations of the experimental data; however, the fitted rate constants were poorly constrained, particularly for the PSO model, because the adsorption reached a near plateau by the first measured time point (5 min). Therefore, the present kinetic data do not allow unambiguous discrimination between the PFO and PSO models. The PSO model is consequently considered here as an empirical description of the observed kinetic behaviour rather than as evidence for a specific adsorption mechanism. In particular, the PSO model does not, by itself, establish electron transfer or chemisorption as the rate-determining step [27]. The adsorption reached a near plateau by the first measured time point (5 min), with no appreciable increase in q_e_ at the subsequent measurement times under the investigated conditions. However, because the adsorption behaviour before 5 min was not resolved experimentally, the present data do not allow the initial rate or the rate-limiting transport step to be established.

The very high R^2^ values obtained for the nonlinear fits (Table 7) should be interpreted with caution because the experimental data reach a near plateau by 5 min. Consequently, the rate constants are poorly constrained, and the high goodness-of-fit values do not provide sufficient evidence to distinguish between the PFO and PSO kinetic equations.

## 4. Conclusions

Among the investigated activation conditions, the best-performing conditions for physical activation were CO_2_, 1000 °C, 180 min, and low loading, resulting in a specific surface area of 712 m^2^·g^−1^ (sample S7). Fractal analysis using the Frenkel–Holsi–Hill model revealed high surface roughness (D_ads2_ = 2.96) and good pore connectivity (D_des_ ≈ D_ads2_). The resulting sorbent exhibits a high PCT capacity for physically activated materials: 629 mg·g^−1^ (neutral medium) and 718 mg·g^−1^ (pH 5). The adsorption is consistent with predominantly weak physical interactions (E < 8 kJ·mol^−1^), with π–π interactions potentially contributing to PCT adsorption, and the pseudo-second-order equation provides a close empirical description of the observed kinetic data. Increasing SSA was accompanied by a change in the relative quality of Langmuir and Freundlich fits; however, these fitting results alone do not demonstrate a transition from a heterogeneous to a homogeneous surface. Thus, pyrolytic carbon black is an environmentally safe and fast-acting sorbent for the removal of pharmaceuticals from water.

Future studies should focus on the further development of methane pyrolysis-derived carbon sorbents through alternative physical activation strategies, particularly steam activation, and comparison with the CO_2_ activation route used in the present study. Such studies may provide additional control over the pore structure and surface properties of the resulting carbon materials. This approach could expand the application of methane pyrolysis-derived carbon beyond paracetamol removal and contribute to the development of multifunctional sorbents for wastewater treatment.

## Figures and Tables

**Table 1:** Dependence of the change in pyrolytic carbon black sample mass on the activation process parameters (crucible volume 162 cm^3^).

Sample Number	Form of Pyrolytic Soot [22]	Initial Mass, g	Activation Temperature, °C	Duration of Activation, min	Mass Loss, wt.%
S1	powder	17.87	800	30 (N_2_) and 60 (CO_2_)	0.07
S2	powder	16.09	1000	30 (N_2_) and 60 (CO_2_)	7.00
S3	powder	13.07	1000	30 (N_2_) and 60 (CO_2_)	18.10
S4	powder	4.67	1000	30 (N_2_) and 60 (CO_2_)	42.40
S5	film	8.76	1000	180 (CO_2_)	7.00
S6	powder	5.46	900	180 (CO_2_)	35.04
S7	powder	5.65	1000	180 (CO_2_)	51.64

**Table 2:** Texture characteristics of the samples.

Sorbent Number	SSA, m^2^·g^−1^	Micropore Volume, cm^3^·g^−1^	Average Pore Radius, nm	Total Pore Volume, cm^3^·g^−1^
S1	3.6	0.001	2.7	0.01
S2	18.5	0.008	1.7	0.03
S3	12.4	0.005	1.7	0.03
S4	75.2	0.034	1.7	0.06
S5	113.1	0.050	1.7	0.09
S6	41.5	0.021	1.5	0.04
S7	712.8	0.296	1.7	0.44

**Table 3:** Results of fractal analysis using the FHH model.

Sorbent Number	Adsorption						Desorption		
	A_ads1_	D_ads1_	R^2^	A_ads2_	D_ads2_	R^2^	A_des2_	D_des_	R^2^
S1	−0.641± 0.012	2.36	0.999	−0.398± 0.018	2.60	0.992	−0.403 ± 0.019	2.59	0.971
S2	−0.256 ± 0.005	2.74	0.999	−0.191± 0.003	2.81	0.999	−0.198 ± 0.008	2.80	0.974
S3	−0.363 ± 0.009	2.64	0.997	−0.232 ± 0.006	2.77	0.997	−0.203± 0.003	2.79	0.996
S4	−0.183± 0.011	2.83	0.986	−0.070 ± 0.003	2.93	0.993	−0.054 ± 0.007	2.95	0.997
S5	−0.239 ± 0.008	2.76	0.995	−0.117 ± 0.008	2.88	0.983	−0.0412 ± 0.0016	2.96	0.977
S6	−0.244 ± 0.005	2.76	0.998	−0.143 ± 0.005	2.86	0.994	−0.081 ± 0.004	2.92	0.971
S7	−0.218 ± 0.016	2.78	0.978	−0.039 ± 0.004	2.96	0.959	−0.0247 ± 0.0010	2.98	0.975

**Table 4:** Parameters obtained by fitting PCT adsorption isotherms to the Langmuir, Freundlich, and Dubinin–Radushkevich models.

Parameters	Sample						
	S1	S2	S3	S4	S5	S6	S7
The Langmuir model							
q_max_, mg⋅g^−1^	-	65	-	49	**23**	**110**	**629**
K_L_, L⋅mg^−1^	-	0.003	-	0.03	**0.20**	**0.22**	**0.13**
R^2^	0.989	0.951	0.986	0.822	**0.973**	**0.978**	**0.992**
The Freundlich model							
K_F_, mg⋅g^−1^	0.027	**0.88**	**0.04**	**7.39**	9.38	34.14	-
n	0.88	**1.66**	**0.91**	**3.17**	6.07	4.6	-
R^2^	0.994	**0.978**	**0.988**	**0.959**	0.898	0.880	0.028
The Dubinin–Radushkevich model							
K, mol^2^⋅kJ^−2^	0.12	0.06	0.12	0.02	0.013	0.016	0.03
E, kJ⋅mol^−1^	2.04	2.92	2.05	4.5	6.15	5.59	4.09
R^2^	0.997	0.954	0.983	0.922	0.927	0.923	0.997

**Table 5:** Parameters of the Langmuir, Freundlich, and Dubinin–Radushkevich models.

Isotherm Type	Parameters	pH 5	pH 9
Langmuir	q_max_, mg⋅g^−1^	718	624
	K_L_, L⋅mg^−1^	0.08	0.05
	R^2^	**0.969**	**0.980**
Freundlich	K_F_, mg⋅g^−1^	-	-
	n	-	-
	R^2^	0.108	0.155
Dubinin–Radushkevich	K, mol^2^⋅kJ^−2^	0.064	0.032
	E, kJ⋅mol^−1^	2.795	3.953
	R^2^	**0.969**	**0.980**

**Table 6:** Parameters of the pseudo-first-order, pseudo-second-order, and intraparticle diffusion models.

Isotherm Type	Parameters	pH 5	pH 9
Pseudo-first-order kinetic (PFO)	q_e_, mg⋅g^−1^	243	165
	k_1_, min^−1^	0.00028	0.00049
	R^2^	0.539	0.374
Pseudo-second-order kinetic (PSO)	q_e_, mg⋅g^−1^	**476**	**459**
	k_2_, g⋅mg^−1^⋅min^−1^	**0.0263**	**0.0308**
	R^2^	**1**	**1**
Intraparticle diffusion (IPD)	C, mg⋅g^−1^	475	459
	K_diff_, g⋅mg^−1^⋅min^−0.5^	-	-
	R^2^	0.565	0.389

**Table 7:** Parameters obtained from nonlinear fitting of the PFO and PSO kinetic models.

Isotherm Type	Parameters	pH 5	pH 9
Pseudo-first-order kinetic (PFO)	q_e_, mg⋅g^−1^	475	458
	k_1_, min^−1^	0.00046	0.00074
	R^2^	0.999	0.999
	χ^2^	3.22616	0.367
Pseudo-second-order kinetic (PSO)	q_e_, mg⋅g^−1^	**476**	**459**
	k_2_, g⋅mg^−1^⋅min^−1^	**0.0234**	**0.0342**
	R^2^	**0.999**	**0.999**
	χ^2^	**1.718**	**1.718**

## Data Availability

The original contributions presented in the study are included in the article material, further inquiries can be directed to the corresponding author.
